# Electrocatalytic and Magnetic Properties of Porous Iron Phosphide Nanorods

**DOI:** 10.1021/acsaem.5c02386

**Published:** 2025-11-05

**Authors:** Shubham Sharma, Sharad Puri, Resham Shrestha, David N. McIlroy, Julius de Rojas, Ali Kaan Kalkan, Yolanda Vasquez

**Affiliations:** † Department of Chemistry, 7618 Oklahoma State University, Stillwater, Oklahoma 74078, United States; ‡ Department of Physics, 7618 Oklahoma State University, Stillwater, Oklahoma 74078, United States; § Department of Mechanical and Aerospace Engineering, 7618 Oklahoma State University, Stillwater, Oklahoma 74078, United States

**Keywords:** iron phosphide, porous nanorods, synthesis, earth abundant catalyst, magnetism, electrocatalyst, HER, water-splitting

## Abstract

Nanoscale transition metal phosphide systems hold significant technological potential due to their distinctive optoelectronic properties, high catalytic activity, superparamagnetism, and high diffusion coefficient of Na/Li ions. However, attempts to synthesize phase-pure FeP, a promising electrocatalyst, have utilized expensive and/or highly reactive phosphide precursors such as tri-*n*-octylphosphine (TOP), white phosphorus (P_4_), tris­(trimethylsilyl)­phosphine (P­(TMS)_3_), and tri-*n*-butylphosphine. These methods often require high temperatures and/or multistep reaction processes. Here, to address these limitations, we present a solution-based synthesis method to produce phase-pure FeP nanoparticles. In this synthesis, we react iron oxyhydroxide (β-FeOOH) as a cost-effective, environmentally friendly, and air-stable source of iron with tris-diethylaminophosphine P­(NEt_2_)_3_ as a phosphorus source at 280 °C. The resulting FeP is formed in a porous nanorod morphology. The particles were characterized by TEM and XPS. Magnetic measurements of the phase-pure FeP nanoparticles indicate paramagnetic behavior, contrasting the antiferromagnetic behavior observed in bulk FeP. In addition to their unique magnetic properties, these porous FeP nanorods demonstrate promising HER performance, achieving an overpotential of 267 mV at a geometric current density of −10 mA cm^–2^ in acidic media, with stable electrocatalytic activity maintained for up to 12 h at −50 mA cm^–2^. This study represents the first documented low-temperature, time-efficient, solution-based thermal decomposition method for synthesizing phase-pure FeP nanoparticles, using tris­(diethylamino)­phosphine P­(NEt_2_)_3_ and iron oxyhydroxide (β-FeOOH) as sources of phosphorus and iron, respectively, at 280 °C.

## Graphical abstract

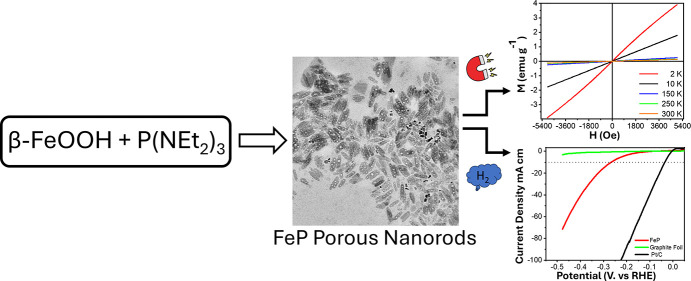

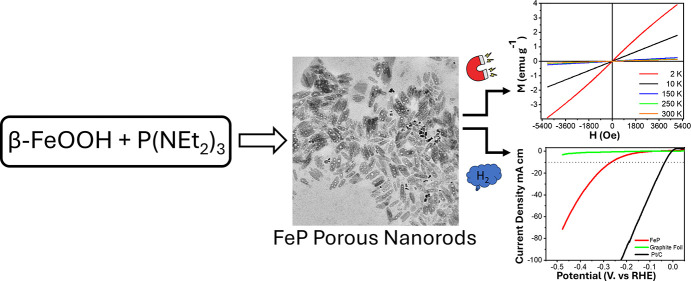

## Introduction

Transition metal phosphides (TMPs) have garnered significant interest in the fields of magnetism and electrocatalysis, as their interatomic spacing and anion electronegativity fall in an intermediate range between those of metals and oxides.^1,2^ Bulk TMPs exhibit a wide range of interesting properties such as superconductivity, thermoelectricity, ferromagnetism, magnetoresistance, and semiconducting behavior.^3–6^ In recent years, TMPs have gained significant attention as electrocatalysts due to their inherent electrical conductivity and stability.^7–10^ Specifically, interest in iron phosphide (FeP) nanostructures has been growing rapidly due to their multifunctional physical and chemical properties. FeP exhibits unique electronic, magnetic, and electrocatalytic behaviors, including metallic conductivity, tunable magnetic ordering, and efficient electrocatalytic activity for hydrogen evolution reactions (HERs).^11–16^ Moreover, phase-pure FeP nanostructures with hierarchical porosity provide a wide range of pore size distributions (PSDs), improve mass transport, and expose more active sites for electrocatalysis. Besides, these structural features can also influence magnetic properties through surface anisotropy and size-domain effects.^17,18^ However, the relationship between their nanoscale structure and catalytic or magnetic behavior remains to be explored. Additionally, earth-abundant and low-cost FeP has exhibited activity toward the HER comparable to that of noble metal catalysts, along with the ability to reduce CO_2_ into oxygenated single- and multicarbon products.^19,20^

Research on the synthesis of nanostructured, phase-pure iron phosphides has been relatively limited compared with the other 3d TMPs, such as cobalt and nickel. This is mainly because iron-phosphide nanostructures display a range of compositions, including metastable phases, such as Fe_3_P, Fe_2_P, FeP_2_, and FeP_4_, along with their nonstoichiometric phases.^21^ Furthermore, traditional sources of Fe and P are highly reactive, air-sensitive, and toxic and require stringent safety precautions. Traditionally, wet-chemistry synthetic methods for iron phosphides have employed commercially accessible iron sources such as Fe nanoparticles, Fe­(CO)_5_, Fe­(acac)_3_, Fe­(N­(SiMe_3_)_2_)_3_, and (η_4_-cyclohexadiene) irontricarbonyl, along with phosphorus sources like tri-*n*-octylphosphine (TOP), ditertbutylphosphine, tristrimethylsilylphosphine, white phosphorus, phosphine gas, and trioctylphosphine oxide (TOPO).^3,6,11,22–25^ However, a common drawback among these synthetic approaches is the use of expensive iron and phosphorus sources that are prone to autoignition when exposed to air, require prolonged reaction times (4–12 h), high reaction temperatures, and generate environmentally concerning byproducts that limit product scalability.^26,27^ Additionally, commonly used phosphorus sources such as TOP have a propensity to form carbide side products, exhibit batch-to-batch variability, and require elevated temperatures (>350 °C) to cleave the P–C bond (264 kJ mol^–1^) to generate the active species, thereby motivating the exploration of alternative synthetic pathways.^6,26,27^

Recently, the development of aminophosphine-based ((R_2_N)_3_P) protocols has emerged as cost-effective, nonhazardous, and convenient precursors for the synthesis of TMP nanosystems under ambient conditions.^25^ Moreover, weaker P–N bonds (195–200 kJ mol^–1^) lower the activation barrier for bond cleavage and facilitate the in situ generation of reactive P species under milder conditions. An aminophosphine-based synthesis is now widely used for the fabrication of the indium phosphide (InP) nanoparticles,^25,28–31^ and has recently been extended to Ni, Co, and Cd-based metal phosphide nanocrystals. However, its application to other phosphide materials, such as FeP, remains unexplored.

In this work, the nonpyrophoric aminophosphine tris­(diethylamino)­phosphine P­(NEt_2_)_3_ is used as a phosphorus precursor to achieve the direct, one-pot synthesis of phase-pure FeP porous nanorods.^25^ These porous nanorods are distinctive in that more than 91% of the particles possess a uniform symmetry, size, and structure. To the best of our knowledge, only one literature report has used tris­(diethylamino)­phosphine as a phosphorus source for FeP synthesis; however, that study employed a two-step phosphorization process at a high temperature (360 °C) using iron pentacarbonyl (Fe­(CO)_5_) as the iron source.^32^ In contrast, the present method achieves FeP formation via a one-pot synthesis by injecting P­(NEt_2_)_3_ into a mixture of iron oxyhydroxide (β-FeOOH) and oleylamine (OLA) at 280 °C. Previous mechanistic studies on P­(NMe_2_)_3_-based hot-injection synthesis highlight the critical role of oleylamine as a coordinating solvent and ligand. Protic amines (e.g., oleylamine, dodecylamine) release labile protons (H^+^), which, as reported in prior studies on P­(TMS)_3_-based InP QDs synthesis,^33,34^ facilitate the hydrolysis of P­(NEt_2_)_3_, generating highly reactive phosphine (PH_3_). The in situ formation of PH_3_ induces rapid nucleation through its reaction with metal–oleylamine complexes, leading to the formation of phosphide materials.^28^ Additionally, the FeP porous nanorods exhibit paramagnetic behavior, unlike the antiferromagnetism characteristic of bulk FeP. These particles were also evaluated for their electrocatalytic activity toward the HER and achieved an overpotential of 267 mV at −10 mA cm^–2^ in acidic media, maintaining stability for 12 h at −50 mA cm^–2^.

## Experimental Section

### Materials

Ferric chloride hexahydrate (FeCl_3_·6H_2_O, 98% ACS grade), 50% (w/v) poly­(ethylenimine) solution (PEI, MW 750,000), and oleylamine (C_18_H_35_NH_2_) (70% technical grade) were purchased from Sigma-Aldrich (St. Louis, MO). Tris-diethylaminophosphine, P­(NEt_2_)_3_, (97%) was purchased from Thermo Scientific. Anhydrous ethyl alcohol (200 proof, absolute, ACS/USP grade) and hexane (ACS/USP grade) were purchased from Pharmco (Brookfield, CT). Transmission electron microscopy (TEM) Cu grids (carbon-coated, 200 mesh) were purchased from Electron Microscopy Sciences (Hatfield, PA).

### Synthesis of FeP Porous Nanorods

All glassware was dried in an oven at 150 °C overnight prior to use. Iron oxyhydroxide (β-FeOOH) nanoneedles were synthesized using methods described in our previous work.^35^ The resulting β-FeOOH nanoneedles had *l* = 87 ± 15 nm and *w* = 56 ± 9 nm. A mass of 0.100 g of β-FeOOH and 3.00 mL of oleylamine were degassed at 120 °C under vacuum for 2 h. The solution was heated to the injection temperature of 200 °C under an argon atmosphere. Upon reaching the injection temperature,1.00 mL of P­(NEt_2_)_3_ was swiftly injected into the mixed solution of β-FeOOH and oleylamine. The reaction temperature was then increased to 280 °C, and the temperature was maintained for 60 min. The resultant FeP nanoparticles were isolated by precipitation with 10–15 mL of hexane and centrifuged at 8000 rpm for 2 min in a centrifuge tube to isolate the solid particles. The black solid was subsequently washed several times with excess ethanol and chloroform until the supernatant became clear.

### Characterization Techniques

The morphology and size distribution of the synthesized FeP particles were analyzed using a JEOL JEM 2100 TEM operating at an accelerating voltage of 200 kV and a beam current of 102 μA. The samples for TEM were prepared by casting a dilute solution of FeP nanoparticles in chloroform on a Cu TEM grid (carbon-coated, square mesh, 200) and drying under vacuum. The crystalline phase of the FeP nanoparticles was analyzed using a Rigaku SmartLab (II) diffractometer with a Cu Kα radiation source (λ = 1.54 Å). The wide-scan angle was varied from 3° to 90°(2θ) at a scan rate of 2°/min. X-ray photoelectron spectroscopy (XPS) was performed at room temperature in an ultrahigh vacuum chamber with a base pressure of 7.6 × 10^–10^ Torr. XPS spectra were obtained using the Mg Kα emission line from a dual-anode X-ray source (PREVAC XR 40B-EC) operated at 345 W with an incident angle of 54.7° and normal emission. The kinetic energy of the photoelectrons was collected and analyzed with an EA 125-hemispherical electron energy analyzer with a resolution of 0.025 eV. ATR-FTIR spectra were recorded on a Thermo Scientific Nicolet (Is50FT-IR) instrument. The textural properties of the synthesized FeP nanoporous rods were measured by N_2_-sorption analysis using a Quantachrome AUTOSORB-1 (AS1-11). The samples were degassed at 150 °C for 24 h under vacuum before the analysis. Specific surface area was estimated by applying the BET model in a pressure range of *P*/*P*_0_ of 0.05–0.30. The Barrett–Joyner–Halenda (BJH) method was used to calculate the PSD using Kernel: “N_2_ at 77 K on carbon (cylinder, Pores, QSDFT equilibrium model).” The pore volume was calculated from the uptake at a relative pressure of *P*/*P*_0_ = 0.99. The post-HER H_2_SO_4_ electrolyte sample was analyzed by using a SPECTRO ARCOS inductively coupled plasma optical emission spectrometer (ICP-OES) at the Soil, Water, and Forage Analytical Laboratory. The magnetic properties of the FeP-porous nanorod were probed using a Quantum Design DynaCool Physical Properties Measurement System (PPMS) equipped with the vibrating sample magnetometer (VSM) option. Samples for magnetic characterization were prepared from nanoparticle material placed in polycarbonate capsules and loaded onto a standard brass half-tube. Zero-field-cooled (ZFC) and field-cooled (FC) measurements were performed. In a ZFC measurement, the sample is cooled down from temperature (300 K) to a minimum temperature (2 K) with no magnetic field applied; a field is then applied, and the magnetization measured as the temperature is increased to 300 K. A FC measurement follows the same procedure, except that a magnetic field is applied during both cooling and heating sweeps.

### Electrochemical Experiments

Electrochemical measurements were conducted using a Gamry potentiostat (Interface 1000-11122A) electrochemical workstation. A standard three-electrode setup was employed using a graphite foil-based working electrode (1 × 1 cm^2^), a graphite rod as the counter electrode, and a double-junction silver/silver chloride (Ag/AgCl) reference electrode. The graphite-foil-based working electrode was fabricated by preparing a slurry containing FeP porous nanorods as the active material (70 wt%, 23.3 mg), conducting carbon (Super P, Alfa Aesar, USA, 15 wt%, 5.0 mg), poly­(vinylidene fluoride) as a binder (Thermo Scientific, USA, 15 wt%, 5.0 mg), and *N*-methylpyrrolidone (NMP, Acros Organics, USA, 90 μL) as the solvent. The slurry mixture was magnetically stirred for 24 h at room temperature (25 °C) to improve uniformity. A working electrode with a mass loading of 0.60 mg/cm^2^ was prepared by drop-casting the slurry onto graphite foil and drying it overnight at 50 °C. All potentials in this study were referenced to the reversible hydrogen electrode (RHE) using the relation *E*_RHE_ = *E*_Ag/AgCl_ + 0.197 V + 0.059pH. The HER performance of the as-synthesized FeP porous nanorod electrodes was evaluated by linear sweep voltammetry (LSV), electrochemical impedance spectroscopy (EIS), and chronopotentiometry. The double-layer capacitance was measured by using cyclic voltammetry (CV).

## Results and Discussion

In a typical aminophosphine-based synthesis of FeP porous nanorods, iron oxyhydroxide (β-FeOOH) nanoneedles were dissolved in a primary amine solvent (e.g., oleylamine). The reaction mixture is degassed at elevated temperature and then heated to 200 °C, and tris­(diethylamino)­phosphine, P­(NEt_2_)_3_, is then swiftly injected. The reaction temperature is subsequently increased to 280 °C and maintained for 1 h. Figure 1a depicts the XRD pattern of the synthesized FeP, which corresponds well to the indexed pattern for orthorhombic FeP (COD #1528058), with peak broadening attributed to the nanoscale crystalline domain size. The distinct peaks observed at 32.9°, 37.3°, 47.1°, 48.6°, and 50.6° are assigned to the diffraction of (011), (111), (220), (211), and (130) of the orthorhombic FeP crystal planes, respectively. Notably, no other diffraction peaks, attributable to either β-FeOOH or iron phosphate, were observed. This finding confirms the conversion of β-FeOOH to FeP.^6,36^ Furthermore, to gain insight into the structural evolution, we systematically varied the TDP amount while keeping all other synthesis conditions constant (β-FeOOH: 0.1 g; reaction temperature: 280 °C; duration: 1 h). The pXRD results revealed that lower TDP volumes (0.1–0.3 mL) favored the coexistence of Fe-rich metastable Fe_2_P and FeP phases, whereas increasing the TDP amount promoted the formation of phase-pure FeP. This suggests that a higher concentration of reactive phosphorus species facilitates the conversion of the metastable Fe_2_P intermediate to the stable FeP, which is consistent with the previous findings. However, upon increasing the volume (>1 mL), no peaks were observed in the pXRD pattern (Figure S1). The phase purity of FeP was further evaluated via structural refinement using the Rietveld method. The crystallographic parameters obtained from the refinement are shown in Figure S2 and a detailed summary in Table S1. Figure S3 shows the crystallographic arrangement of FeP porous nanorods, confirming an orthorhombic crystal structure consistent with the Pna2_1_ space group. The synthesized FeP porous nanorods were further investigated by FTIR spectroscopy and XPS. FTIR spectra in Figure 1b show characteristic absorption bands of the tris-diethylaminophosphine (TDP) in the wavenumber range of 2800–3000 and 1450–1700 cm^–1^, corresponding to −C–H stretching and −N–P stretching, respectively. This observation indicates that TDP binds to the surface of the particles and functions as a capping agent.^37,38^ Additionally, the peak around 1100 cm^–1^ is attributed to an oxidized phosphorus species, such as triethyl phosphite P­(OC_2_H_5_)_3_ and phosphates (PO_4_^3–^).^38^ Notably, no −Fe–O absorption bands were observed, further confirming the conversion from the β-FeOOH phase to the FeP phase. In-situ hydrolysis of P­(NEt_2_)_3_ generates highly reactive phosphine (PH_3_) that reacts with β-FeOOH nanoneedles to generate FeP. The N_2_-sorption isotherm of resultant FeP porous nanorods measured at 77 K showed a narrow hysteresis loop in the high-pressure region between *P*/*P*_0_ ratios of 0.40 and 0.65, suggesting that the products held a mesoporous structure (Figure 1c). Moreover, the PSD of the FeP porous nanorods revealed the presence of mesopores ranging widely from 2 to 50 nm (Figure 1d). The specific surface area and micropore volume were measured to be 2.817 m^2^ g^–1^ and 0.006 cm^3^ g^–1^, respectively (Table S2). These results confirm that the FeP porous nanorods possess hierarchical mesoporous cavities, which are beneficial for promoting efficient access to active catalytic sites, improving interfacial contact at phase boundaries, and facilitating H_2_ release during the HER.^39^ Furthermore, these hierarchical cavities introduced an enhanced surface anisotropy effect, potentially leading to paramagnetic behavior in contrast to bulk FeP.

**Figure 1:**
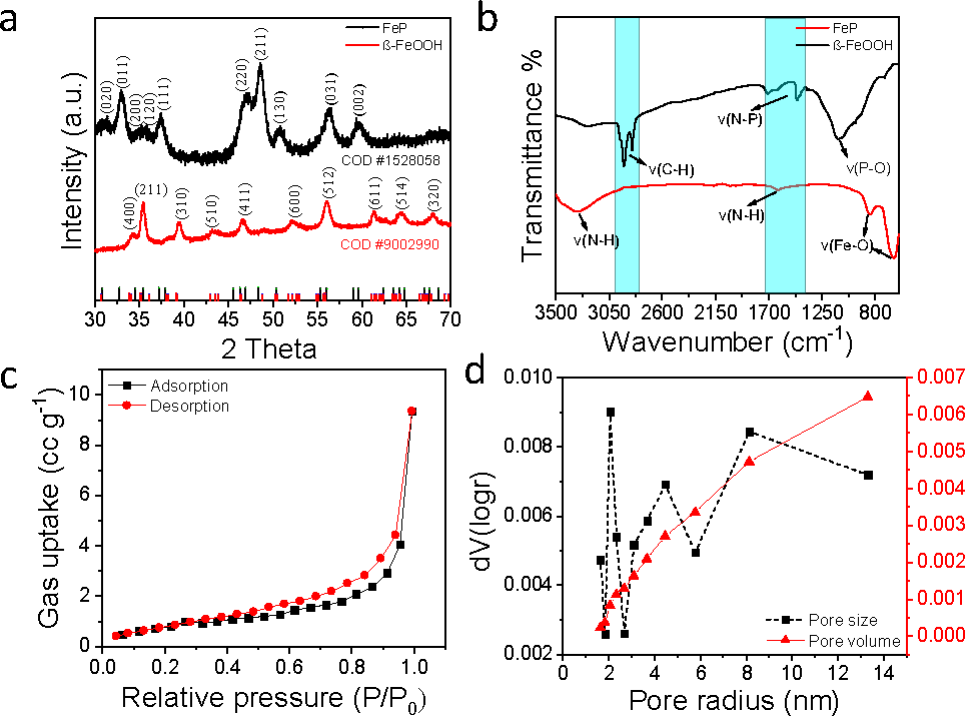
Characterization of FeP porous nanorods synthesized from β-FeOOH nanoneedles and tris-diethylaminophosphine: (a) pXRD pattern of β-FeOOH (COD #9002990) and FeP (COD #1528058); (b) FTIR spectra of β-FeOOH and FeP; (c) N_2_-sorption isotherm; (d) pore size distribution (PSD) and cumulative pore volume of FeP porous nanorods.

XPS was conducted to investigate the electronic structure and chemical environment of the synthesized FeP porous nanorods. As shown in Figure S4, the survey spectrum confirmed the presence of Fe, P, C, and O, with the oxygen attributed to the superficial adsorption and oxidation of the FeP porous nanorods. Figure 2a,b shows the high-resolution XPS core-level spectra for the Fe 2p and P 2p regions of phase-pure FeP porous nanorods, respectively. The Fe 2p spectrum exhibits distinct peaks at 706.9 and 720.1 eV, corresponding to Fe 2p_3/2_ and Fe 2p_1/2_, respectively, which agree with reported values for FeP. Additional peaks at 710.8 and 724.2 eV are attributed to oxidized Fe species on the surface of FeP.^9,10,40^ The high-resolution P 2p spectrum exhibits a peak at 134.5 eV, attributed to oxidized phosphorus species such as phosphite (PO_3_^3–^) and phosphate (PO_4_^3–^), whereas the peaks at 131.7 and 130.1 eV correspond to the P 2p_1__/__2_ and P 2p_3__/__2_ states, characteristic of the FeP phase.^41–43^

**Figure 2:**
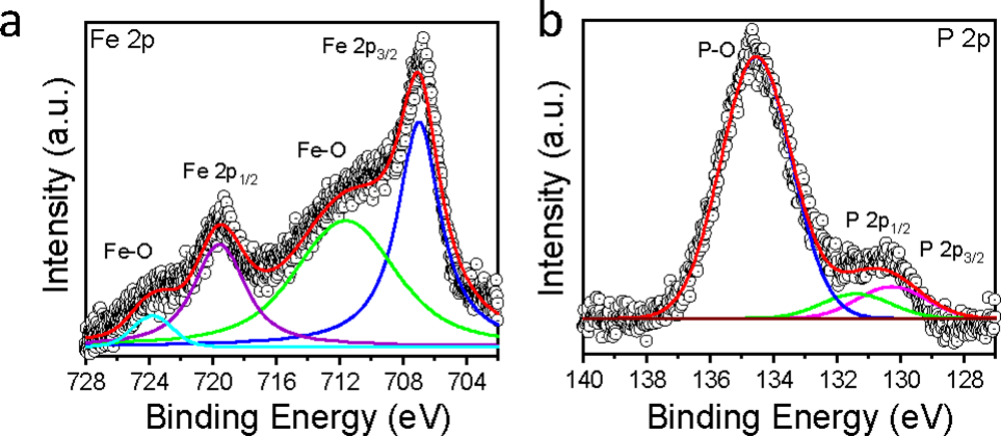
XPS core level spectra for FeP porous nanorods: (a) Fe 2p and (b) P 2p region.

The microstructure of the as-synthesized FeP particles was examined using TEM (Figure 3). The images show that the particles are polycrystalline, predominantly exhibiting a porous nanorod morphology. The internal porosity of the nanorods likely results from structural transformations during β-FeOOH phosphorization. This is attributed to the Kirkendall effect, arising from different diffusion rates of active phosphorus and oxygen species, or partial dehydration of β-FeOOH at 280 °C.^3,44,45^ Additionally, a subpopulation of high-contrast nanospheres was also observed, consistent with results reported by Cossairt et al.^25^ This is possibly due to secondary nucleation growth or isotropic surface interactions (Figure 3a). A high-magnification TEM image provides a closer view of the porous nanorod structure (Figure 3b). Lattice-resolved TEM images of the porous nanorods and high-contrast nanospheres (Figure 3c,d) show lattice spacings of *d*_2__1__0_ = 0.25 nm and *d*_0__2__0_ = 0.29 nm, respectively, while XRD reflects dominant bulk planes ((121), (101), and (111)). This observation suggests that crystal growth in the analyzed region is oriented along the (020) plane.^46,47^ These atomic planes characterize the orthorhombic FeP phase.

**Figure 3:**
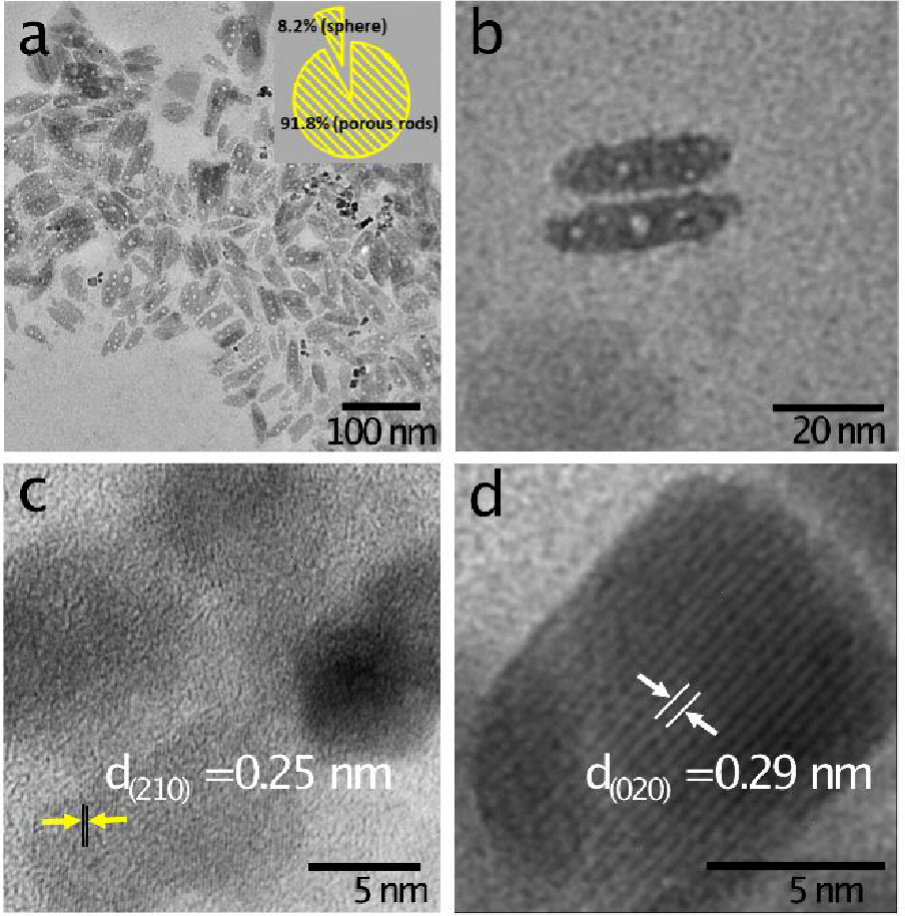
TEM images of FeP nanoparticles. (a) Low-magnification TEM image highlighting the anisotropic morphology, consisting of porous nanorods and nanospheres. (b) High-magnification TEM image providing a view of the porous nanorod structure. (c, d) Lattice fringes of the porous nanorods and nanospheres, showing lattice spacings of *d*_2__1__0_ = 0.25 nm and *d*_0__2__0_ = 0.29 nm, respectively, consistent with the orthorhombic FeP phase.

To probe the effect of nanoscale dimensions on the magnetic properties of FeP nanoparticles, temperature-dependent magnetization measurements were performed by using a VSM magnetometer. Figure 4a shows the DC magnetization plot of the as-synthesized FeP nanoparticles. The magnetization versus temperature for ZFC and FC experiments at different magnetic fields (100 Oe, 1000 Oe, 10,000 Oe) is also presented. No difference was detected between the experimentally measured ZFC and FC states for the synthesized FeP nanoparticles, even when subjected to a field as low as 100 Oe (0.01 T) and temperatures of 2 K.^22^ The overlapping plots are consistent with paramagnetic (Curie–Weiss) behavior and agree with the previously reported literature on FeP nanoparticles^48^ and FeP nanowires.^1,22^ Figures 4b and S5 show the magnetization versus applied magnetic field plots at different temperatures. At 300 K, no measurable magnetization is observed in the magnetization versus field curves. However, as the temperature is reduced to 2 K, a linear increase in magnetization with the applied field is observed, exhibiting a profile characteristic of paramagnetic materials.^48^ The highest magnetization value was 3.90 emu g^–1^ for the FeP nanorod samples at 2 K.^22,49–52^

**Figure 4:**
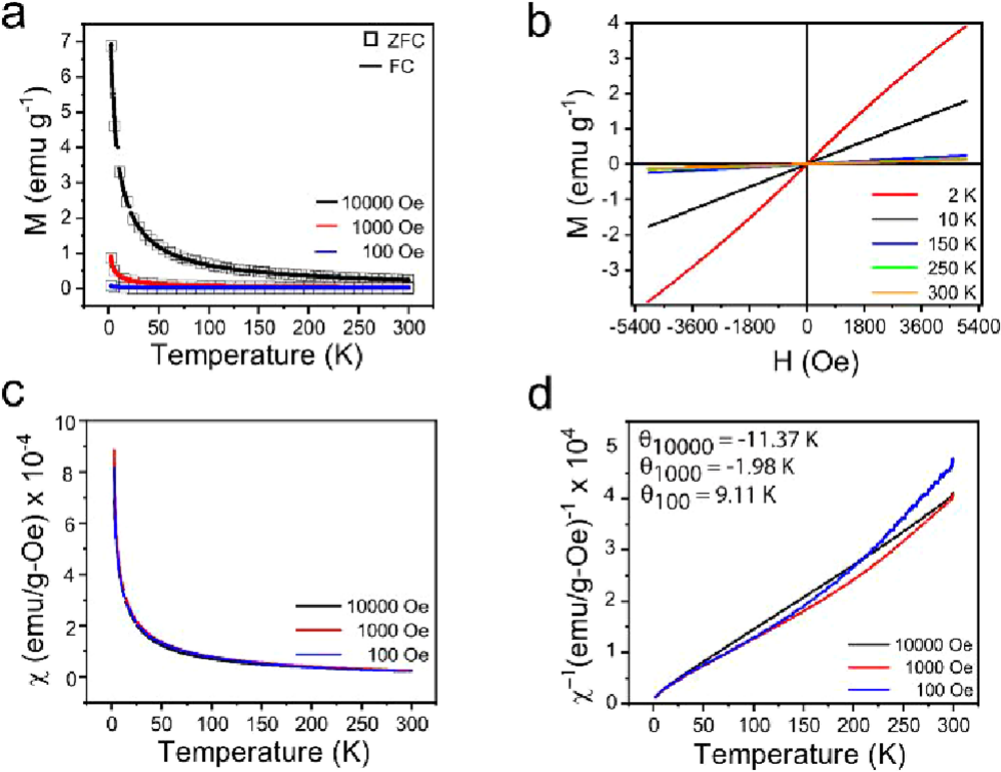
Magnetic characterization of as-synthesized FeP porous nanorods: (a) ZFC/FC curves under applied fields of 10,000 Oe, 1000 Oe, and 100 Oe; (b) temperature-dependent hysteresis (*M*–*H*) loops; (c) temperature-dependent magnetic susceptibility (χ) plots for fields of 10,000 Oe, 1000 Oe, and 100 Oe; and (d) temperature-dependent inverse susceptibility (χ^–1^) plot.

The magnetic properties of the as-synthesized FeP porous nanorods were evaluated by using temperature-dependent magnetic susceptibility (χ), as shown in Figure 4c. In bulk FeP, antiferromagnetic behavior characterized by a double helical magnetic structure is observed below a Néel temperature (*T*_N_) of 115 K.^1^ In contrast, the synthesized FeP porous nanorods exhibit a paramagnetic response consistent with Curie–Weiss behavior between 300 and 50 K, with no distinct magnetic ordering temperature evident. Below 50 K, there is a noticeable increase in the rate of magnetization, as shown in the inset of Figure 4c, as the thermal energy decreases relative to the Zeeman energy. The temperature-dependent plot of inverse susceptibility (χ^–1^) was also obtained for the synthesized FeP nanorod sample, as shown in Figure 4d. The linear extrapolation of the data from the high-temperature region of the (χ^–1^) versus temperature (T) revealed a slight negative Curie–Weiss temperature (θ = −11.37 K and −1.96 K), as shown in Figure 4d, indicating paramagnetism with weak antiferromagnetic correlations. At 100 Oe, a small positive θ (+9 K) was observed, which could be attributed to spin canting effects leading to weak ferromagnetic-like behavior at low fields, as detailed in the fitting shown in Figure S6. Since no magnetic anomalies were observed in the ZFC and FC measurements, nor in the *M*–*H* plot at different temperatures, the increase in magnetization beyond the paramagnetic contribution at lower temperatures could be caused by the incomplete neutralization of antiferromagnetic spins within these nanoparticles.^6,22^ This magnetic behavior observed in FeP resembles that of various antiferromagnetic nanoparticles, including NiO and ferritin.^22,50,53–55^

Additionally, the χ^–1^ v. *T* plots exhibit an increasingly negative curvature above 50 K as the applied magnetic field is decreased rather than an ideal linear trend. The likely origin is a negative, temperature-independent contribution to susceptibility, χ_0_, which can be ascribed to several sources, including core diamagnetism in the sample or the sample holder.^56^ The decrease in χ_0_ (and curvature) with increasing field results from the suppression of diamagnetism with increasing field strength. These findings suggest that the magnetic properties of the FeP particles may arise from nanoscale effects, leading to altered magnetic characteristics compared to bulk FeP. We cannot rule out the possible paramagnetic and diamagnetic contributions from iron oxides or surface stabilizers, respectively. Although our ATR-FTIR and pXRD analyses revealed no observable evidence of iron oxides in the sample, the XPS data indicate the presence of oxidized iron. A more detailed investigation of the magnetic properties is necessary to elucidate the magnetism of these porous nanorods and specifically determine the origin of their paramagnetic behavior in contrast to the bulk material.

FeP is among the most promising, cost-effective, and earth-abundant alternatives to Pt and Pd/PtRu-based electrocatalysts for HER applications, which is attributed to its high electrical conductivity and rapid charge transfer kinetics. Drawing inspiration from these favorable electrocatalytic properties, we also examined the electrocatalytic HER performance of the synthesized FeP porous nanorods, with electrodes prepared by coating FeP nanorods on graphite foils. An electrolyte of 0.50 M H_2_SO_4_ was employed. For comparison, electrocatalytic HER characteristics of bare graphite foil and commercially available Pt/C (20 wt%, Thermo Scientific) electrodes were also measured. Figure 5a shows the polarization curves (*j*–*V* plots) for FeP (mass loading = 0.60 mg cm^–2^), bare graphite foil, and Pt/C in 0.50 M H_2_SO_4_. As expected, Pt/C exhibited superior HER activity with a low overpotential. In contrast, bare graphite foil exhibited limited catalytic activity, demanding an overpotential exceeding 500 mV to achieve a geometric current density of −10 mA cm^–2^. Notably, FeP-coated graphite foil achieved a current density of −10 mA cm^–2^ with a significantly lower overpotential of 267 mV. The HER performance of FeP porous nanorods is comparable to previously reported TMPs and other non-noble metal-based catalysts.^9,10,20,40^ The corresponding Tafel plots are shown in Figure 5b. The Pt/C (20 wt%) shows a lower Tafel slope of 39 ± 1.24 mV/decade, close to the previously reported nanostructured FeP electrocatalysts in the literature.^20,57,58^ The bare graphite electrode shows a significantly higher Tafel slope of 210 ± 2.12 mV/decade, while the FeP porous-nanorod electrode exhibits a Tafel slope of 110 ± 0.72 mV/decade. The relatively low Tafel slope value can be attributed to the efficient charge transfer pathway within the FeP orthorhombic crystal structure, facilitated by the formation of proton- and hydride-acceptor centers as well as the porous structure of nanorods.^59^ EIS for FeP porous nanorods was also conducted and compared with 20 wt% Pt/C, as shown in Figure 5c. The Nyquist plot shows no minuscule semicircle (at lower *Z*’) for the FeP electrocatalyst, suggesting the low interfacial resistance (*R*_ct_) between the electrode and the electrolyte. This low interfacial resistance may be ascribed to the effective diffusion of generated H_2_ through the tubular pores, which prevents blockage of active catalytic sites.^59^ Additionally, these porous networks could enhance the electrolyte-electrode interface area, decreasing the overpotential. The stability of FeP electrocatalysts was evaluated galvanostatically at a current density of 50 mA cm^–2^ (Figure 5d). Within 12 h, a slight decrease is observed in the magnitude of the overpotential, which can be attributed to the exposure of more catalytically active sites on the wetted electrode surface, resulting from the percolation of the electrolyte within the porous structures during the HER.^59^

**Figure 5:**
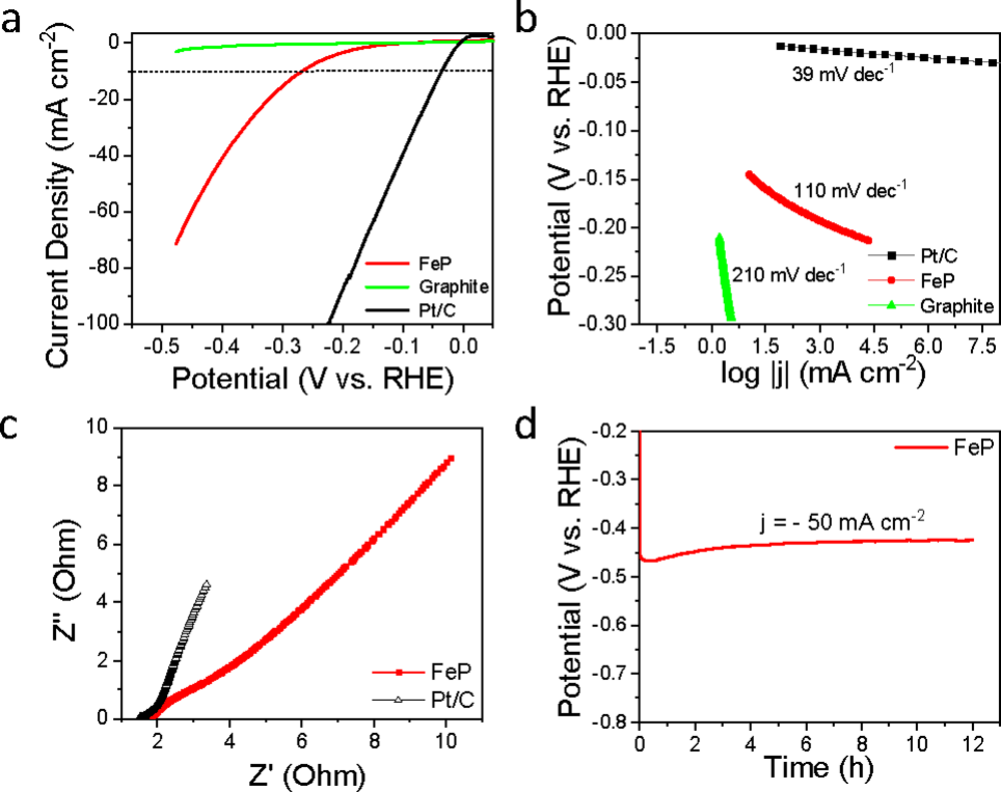
Electrochemical performance of FeP porous nanorods toward HER in 0.50 M H_2_SO_4_: (a) polarization plots of FeP porous nanorods, bare graphite, and Pt/C; (b) Tafel plots of FeP porous nanorods, bare graphite, and Pt/C; (c) Nyquist plots of FeP porous nanorods and Pt/C measured from 10 kHz to 1 Hz at open circuit potential; and (d) chronopotentiometric stability curve of FeP at a constant current density of −50 mA cm^–2^ over 12 h.

Further, to investigate the correlation between the number of active sites and the electrocatalytic HER activity of FeP porous nanorods, double-layer capacitance (*C*_dl_) was quantified through CV measurements at scan rates ranging from 5 to 50 mV s^–1^ within the non-Faradaic region, as illustrated in Figure 6a,b. The experimentally obtained *C*_dl_ value of 26 ± 1.32 μF/cm^2^ indicates that the unique porous nanorod morphology provides a significant electrochemically active surface area (ECSA), which could be a factor in the enhanced HER activity. The FeP porous nanorods demonstrate electrocatalytic HER performance comparable to various iron-based phosphide morphologies in acidic media (Figure 6c); full details are in (Table S3). Furthermore, we performed XPS and FE-SEM analyses on the FeP working electrode, as well as ICP-OES on the 0.5 M H_2_SO_4_ electrolyte, after chronopotentiometry (−50 mA/cm^2^ for 12 h) to gain insight into surface chemistry changes and possible leaching. ICP-OES confirmed Fe and P leaching of 22.73 and 18.19 ppm, respectively (Table S4). XPS showed attenuation of the FeP and PO_4_^3–^ peaks within the ∼10 nm sampling depth, confirming partial leaching. Notably, the significant reduction in Fe 2p_1__/__2_ intensity suggests surface reconstruction, likely involving the formation of a Fe–O–P passive layer or FePO_4_ species (Figure S6).^60^ An image of both the pristine and used electrodes showed robust adhesiveness even after 12 h of chronopotentiometry exposure (Figure S7). FE-SEM confirmed a similar microstructured topography after chronopotentiometry. However, deep microchannels were formed due to the negative potential-driven deformation or percolation of H_2_SO_4_ (Figure S8). The Fe:P stoichiometry is not exactly a 1:1 ratio, likely because EDX measurements were performed on agglomerates rather than individual nanorods and likely due to the presence of surface phosphates in the pristine electrode before chronopotentiometry. EDX mapping confirmed the presence of both Fe and P after testing. Elemental analysis also showed F and S in the pristine electrode, attributed to PVDF binder and graphite foil contamination, respectively. Additionally, Al and Si were observed after chronopotentiometry, likely originating from partial etching of borosilicate glass during the potentiometric experiments in H_2_SO_4_, while the presence of S is attributed to sulfate ions in the electrolyte. The lower Fe atomic % suggests iron leaching during HER, consistent with ICP-OES results (Figure S9).

**Figure 6:**
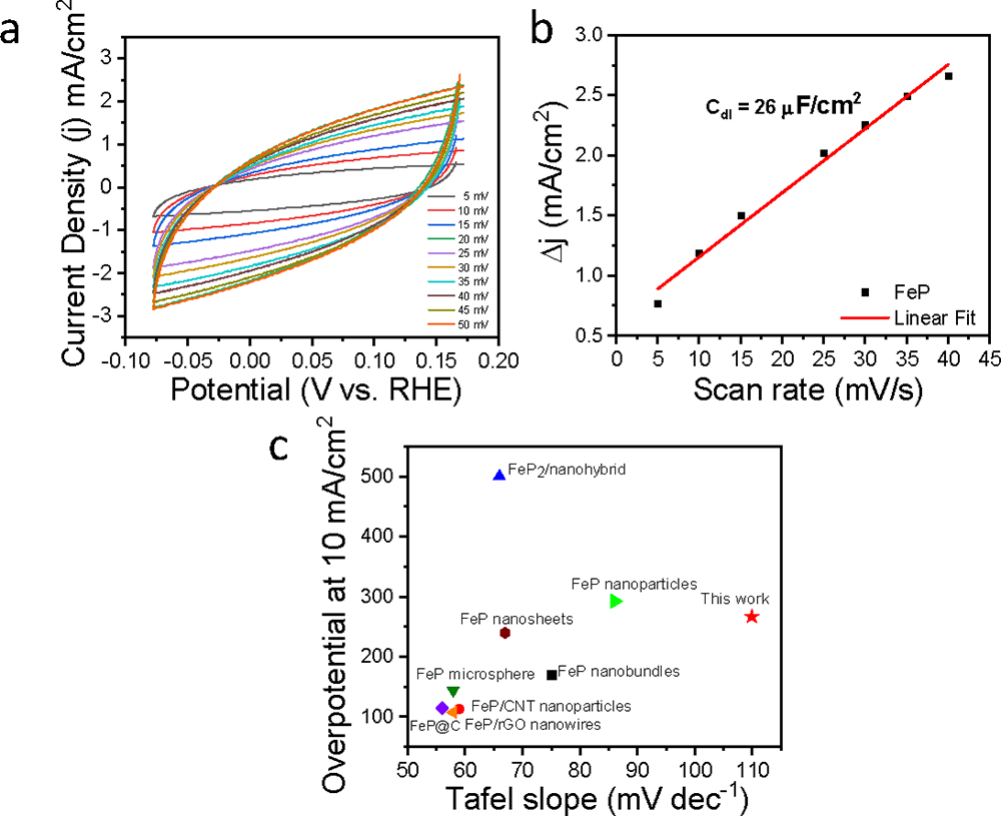
(a) Cyclic voltammetry (CV) profile of FeP nanorods at different scan rates within the non-Faradaic region. (b) Relationship between current density and scan rate used to estimate the double-layer capacitance (*C*_dl_) of FeP nanorod electrodes. (c) Electrochemical HER performance of FeP porous nanorods against previously reported iron-based phosphides in 0.5 M H_2_SO_4_.

## Conclusion

In summary, we have demonstrated a simple and cost-effective method for synthesizing phase-pure iron phosphide porous nanorods. This approach employed a more economical and stable phosphorus source, P­(NEt_2_)_3_, instead of the commonly used TOP or TOPO, in the presence of an amine solvent. Oleylamine was identified as a crucial coordinating solvent that activates P­(NEt_2_)_3_, facilitating the supply of reactive phosphorus species and ultimately leading to the formation of porous FeP nanorods. Moreover, the synthesized FeP porous nanorods exhibited paramagnetic behavior with weak antiferromagnetic interactions, distinct from those of their bulk counterparts. Furthermore, FeP porous nanorods demonstrate efficient HER activity, requiring a 267 mV overpotential for *j* = −10 mA/cm^2^ in 0.5 M H_2_SO_4_, and maintain stability at −50 mA/cm^2^ for 12 h with no performance loss. With these promising results, further studies on the effect of synthetic parameters and detailed characterization of the FeP system are planned in the future to achieve a controlled morphology. This will enable its potential use in various applications, including optoelectronics, energy conversion, and catalysis.

## Supplementary Material



## Notes

PXRD patterns as a function of TDP concentration; Refined structure parameters and crystal structure for as-synthesized FeP porous nanorods; XPS survey spectra; BET surface area analysis report; Enlarged plot of the magnetization versus applied magnetic field; Linear regression fitting profile (χ^–1^) *v* temperature plot; A comparison table of iron-based phosphide electrocatalysts with different morphology in a 0.5 M H_2_SO_4_ electrolyte; Post-HER ICP-OES analysis of the electrolytes; XPS spectra, FE-SEM images, and EDX spectra before and after HER (PDF)

## Abbreviations

TMP: transition metal phosphide
TN: Néel temperature
ZFC: zero field cooling
FC: field cooling
HER: hydrogen evolution reaction
