# Supplementary material for: Electrocatalytic and Magnetic Properties of Porous Iron Phosphide Nanorods
Source: ACS Appl Energy Mater. 2025 Nov 5;8(22):16601–9. doi: 10.1021/acsaem.5c02386 (PMC12648468; doi:10.1021/acsaem.5c02386)
Supplement: Supplementary file 1 [file ae5c02386_si_001.pdf]

Supporting Information

Electrocatalytic and Magnetic Properties of Porous Iron Phosphide Nanorods

Shubham Sharma,[1] Sharad Puri,[2] Resham Shrestha,[1] David N. McIlroy,[2] Julius de Rojas,[2] Ali Kaan Kalkan,[3] Yolanda Vasquez[1,] *

[1]Department of Chemistry, Oklahoma State University, Stillwater, OK 74078, United States

[2]Department of Physics, Oklahoma State University, Stillwater, OK 74078, United States

[3]Department of Mechanical and Aerospace Engineering, Oklahoma State University, Stillwater, Oklahoma 74078, United States

Corresponding Author

***Yolanda Vasquez** yolanda.vasquez@okstate.edu

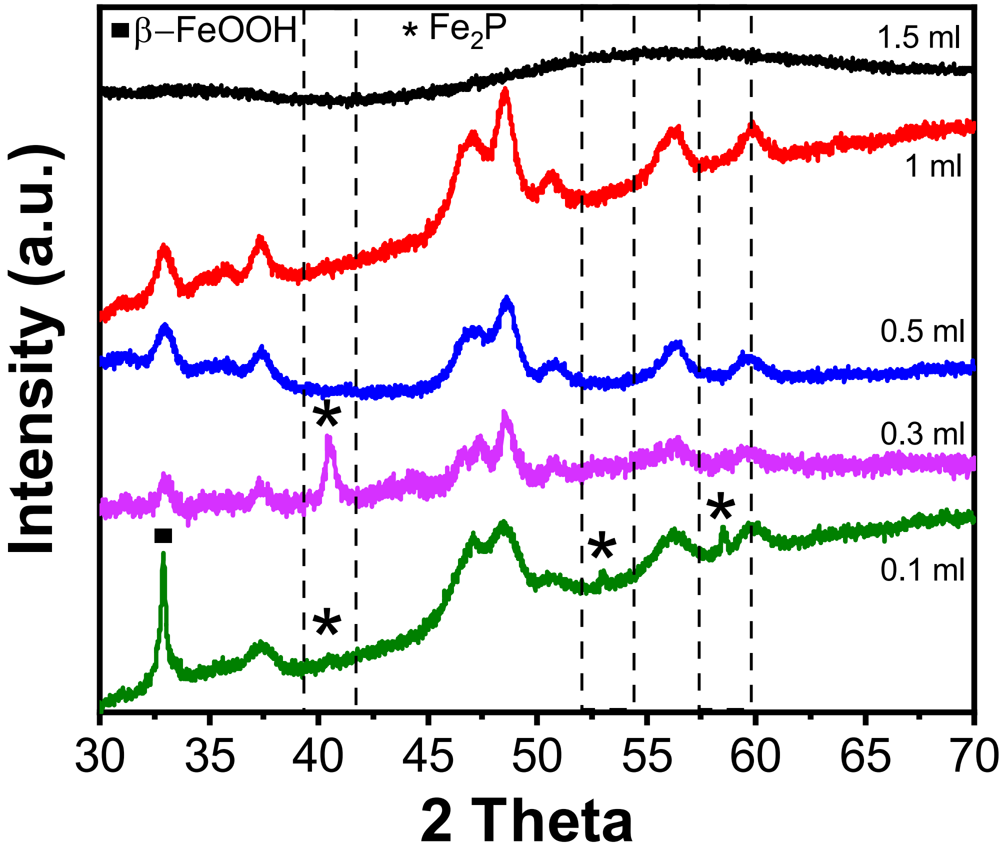

Figure S1. PXRD pattern of the FeP nanoparticles as a function of TDP concentration. All the reactions were performed at 280 °C for 60 minutes.

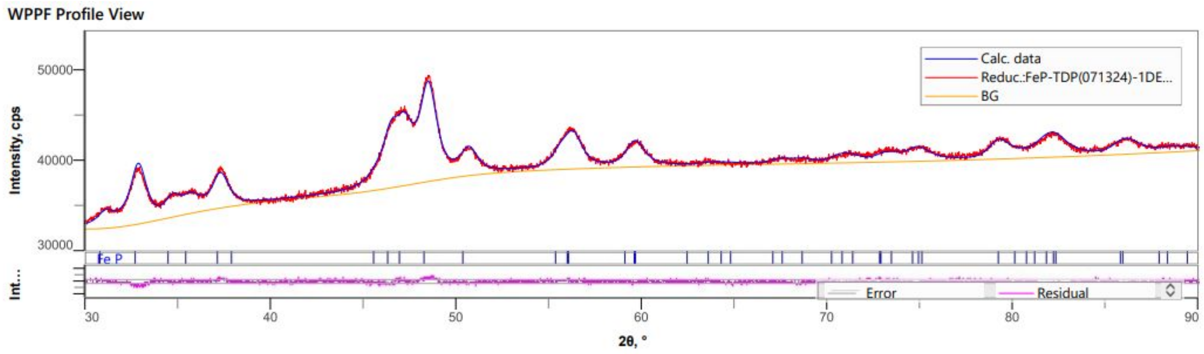

Figure S2. Rietveld refinement profile of FeP porous nanorods. The XRD pattern was fitted and refined using Smart Lab Studio (Rigaku) software.

Table S1. Crystal data and refinement parameters for FeP porous nanorods.

Data	Value
**Chemical Formula**	FeP
**Composition**	FeP
**Z-value**	4.00
**Concentration, wt%**	100.00
**RIR value**	2.334<br>(From structure)
**DB card number**	1528058
**Crystal system**	Orthorhombic
**Space group**	33: Pna21
**a, Å**	5.1888
**b, Å**	5.7821
**c, Å**	3.1088
**α, °**	90.000
**β, °**	90.000
**γ, °**	90.000

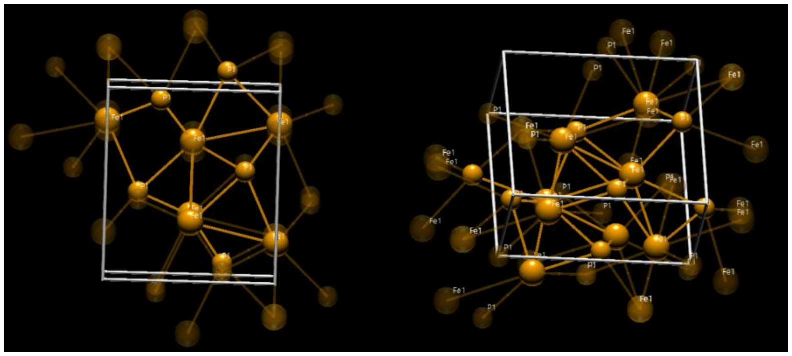

Figure S3. Side view of the FeP crystal structure along the a-axis and corresponding packing structure along the c-axis.

Table S2. Detailed Report of N_2-sorption isotherm measured at 77 K.

Quantachrome AS1Win™ - Automated Gas Sorption Data
Acquisition and Reduction
©1994-2009, Quantachrome Instruments
version 2.11

Analysis | Report
Operator:MatererLab Date:7/11/2025 | Operator:MatererLab Date:7/12/2025
Sample ID: 001 Filename: C:\QCdata\Physisorb\YV_7_11_2025.QPS
Sample Desc: Comment: N2
Outgas Time: 24.0 hrs Outgas Temp: 120.0 °C Sample Weight: 0.2232 g
Analysis gas: Nitrogen (SF) Molec. Wt: 28.0134 g Non-ideality: 6.58e-05 1/mmHg
Analysis Time: 270.0 min Instrument: Autosorb 1 Bath temp.: 77.3 K
Thermal Transpiration: onEff. mol. diameter (D): 3.5400 ÅEff. cell stem diam. (d): 4.0000 mm
t-Method Calc. method: de Boer
BJH/DH method Moving pt. avg.: off Ignoring P-tags below 0.35 P/Po
Adsorbate Nitrogen Temperature 77.350K
Molec. Wt.: 28.013 g Cross Section: 16.200 Å² Liquid Density: 0.808 g/cc
Contact Angle: 0.0 degreesSurf. Tension: 8.850 erg/cm²

Radius	Pore Volume	Pore Surf Area	dV(r)	dS(r)	dV(logr)	dS(logr)
Å	cc/g	m²/g	cc/Å/g	m²/Å/g	cc/g	cc/g
16.4056	2.2679e-04	2.7648e-01	1.2516e-04	1.5258e-01	4.7230e-03	5.7578e+00
18.3898	3.5872e-04	4.1996e-01	6.1187e-05	6.6545e-02	2.5879e-03	2.8145e+00
20.6914	8.2201e-04	8.6777e-01	1.8932e-04	1.8300e-01	9.0095e-03	8.7085e+00
23.3995	1.1196e-03	1.1221e+00	1.0022e-04	8.5661e-02	5.3926e-03	4.6091e+00
26.7709	1.2798e-03	1.2418e+00	4.2449e-05	3.1713e-02	2.6123e-03	1.9516e+00
31.0789	1.6301e-03	1.4672e+00	7.2351e-05	4.6559e-02	5.1671e-03	3.3251e+00
36.7857	2.0863e-03	1.7153e+00	6.9429e-05	3.7748e-02	5.8651e-03	3.1888e+00
44.7007	2.7106e-03	1.9946e+00	6.7419e-05	3.0165e-02	6.9144e-03	3.0936e+00
57.8472	3.3470e-03	2.2146e+00	3.7360e-05	1.2917e-02	4.9401e-03	1.7080e+00
81.4429	4.7212e-03	2.5521e+00	4.5568e-05	1.1190e-02	8.4469e-03	2.0743e+00
133.1045	6.4826e-03	2.8167e+00	2.4074e-05	3.6173e-03	7.1886e-03	1.0801e+00

BJH desorption summary

Surface Area = 2.817 m²/g
Pore Volume = 0.006 cc/g
Pore Radius Dv(r) = 20.691 Å

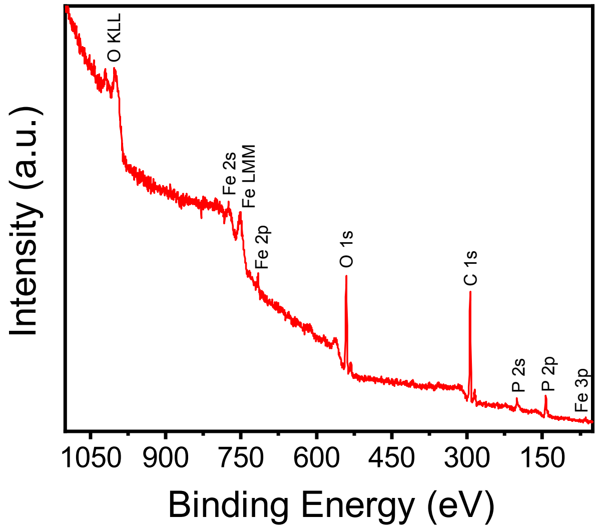

Figure S4. X-ray photoelectron spectroscopy (XPS) survey spectrum of FeP porous nanorods.

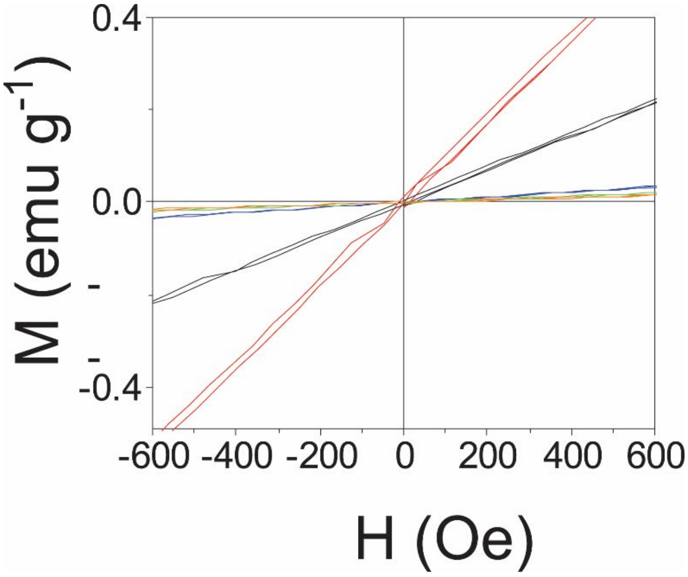

Figure S5. Enlarged hysteresis plot at low applied magnetic fields. Key features include the plots at temperatures of 2 K (red curve) and 10 K (black curve).

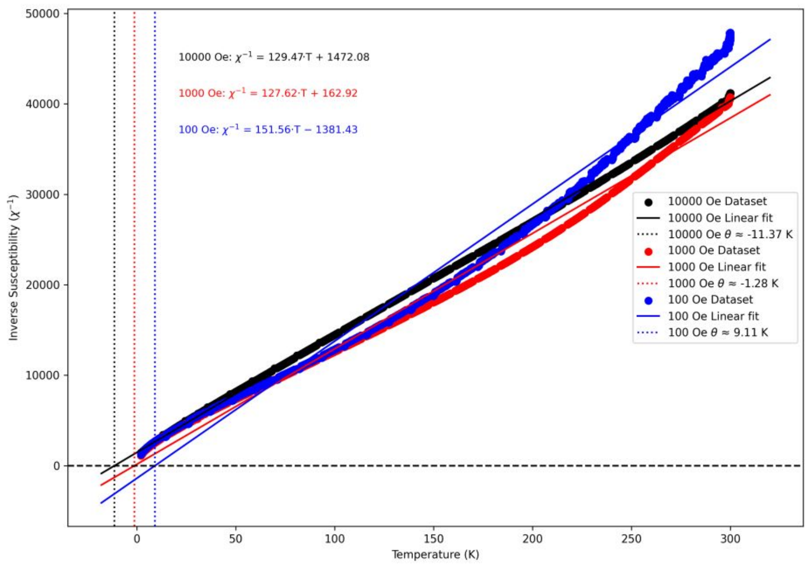

Figure S6. Linear regression fitting of the high-temperature region in the (χ^{-1}) versus temperature plot for determining the Curie–Weiss temperature.

Table S3. HER performance of iron-based phosphide electrocatalyst with different morphology in a 0.5 M H_2SO_4 electrolyte.

**Catalyst**	**Morphology**	**Mass loading (mg/cm^2)**	**Current density (-j) (mA/cm^2)**	**Overpotential at corresponding current density**	**Tafel slope (mV/Dec)**	**Reference**
**FeP**	**Porous-NRs**	**0.60**	**10**	**267**	**110**	**This work**
FeP	Nanobundles	0.85	10	170	75	[1]
FeP/ NCNT	Nanoparticles	0.36	10	113	59	[2]
FeP_2/C	Nanohybrid	0.425	5	500	66	[3]
FeP	Hollow microspheres	0.5	10	144	58	[4]
FeP@C	Hollow microcubes	0.72	10	115	56	[5]
FeP/rGO	Nanowires	0.204	10	107	58	[6]
FeP	Nanoparticles	0.36	10	292	86	[2]
FeP	Nanosheets	0.285	10	240	67	[7]
FeP NP@NPC	Nanoparticles	1.45	10	130	67	[8]
FeP	Nanorods	0.565	10	107	54	[9]
FeP	Nanosheets	0.56	10	129	64	[9]
FeP	Super-structure	0.56	10	66	46	[9]
Fe-Co_xP	Nano-composite	-	10	127	55	[10]
FeP	Nanoparticles	-	10	172	68	[10]
FeP	Nanosphere	0.25	10	256	87	[11]

Table S4. Post-HER analysis of 0.5 M H_2SO_4 (25 mL) electrolyte.

**Lab ID: 2189**	**ICAP-P ppm**	**Fe ppm**
0.5 M H_2SO_4 electrolyte	18.19	22.73

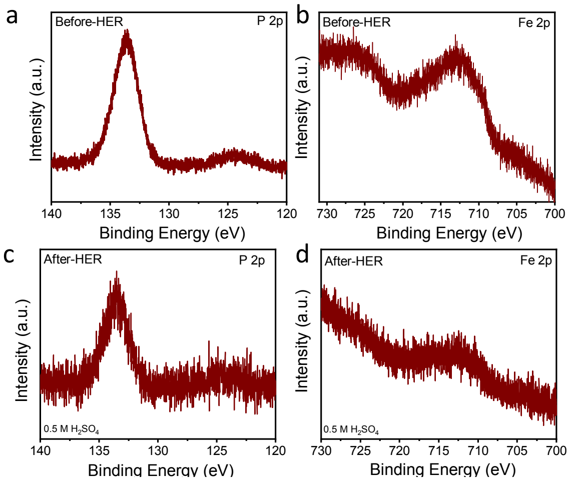

Figure S6. High-resolution XPS spectra of FeP electrodes: (a) P 2p and (b)Fe 2p regions before HER, and (c) Fe 2p and (d) P 2p regions after 12 h testing in 0.5 M H_2SO_4.

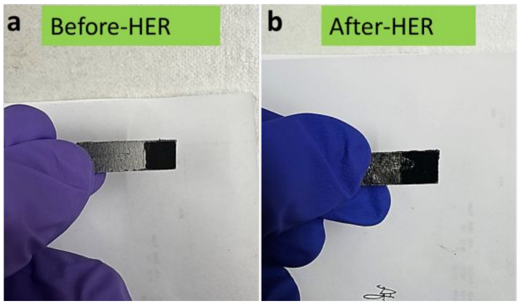

Figure S7. Cross-sectional profiles of the FeP electrode (a) before and (b) after HER testing.

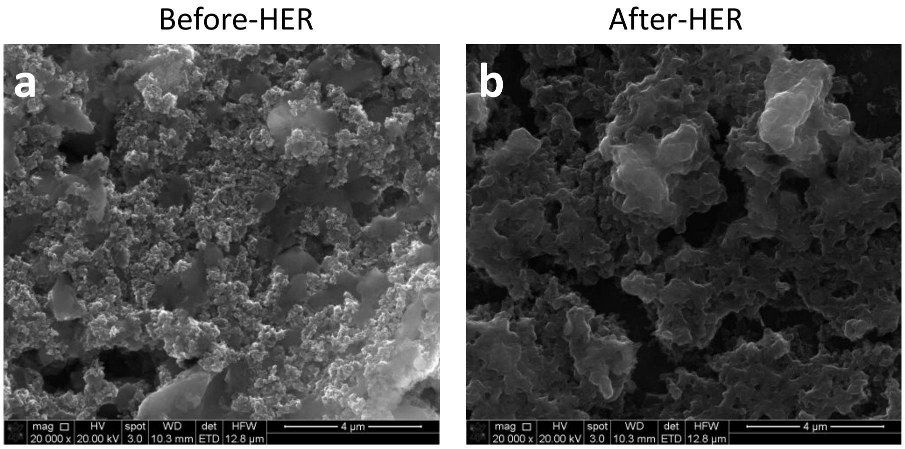

Figure S8. FE-SEM images of the FeP electrode (a) before and (b) after HER testing.

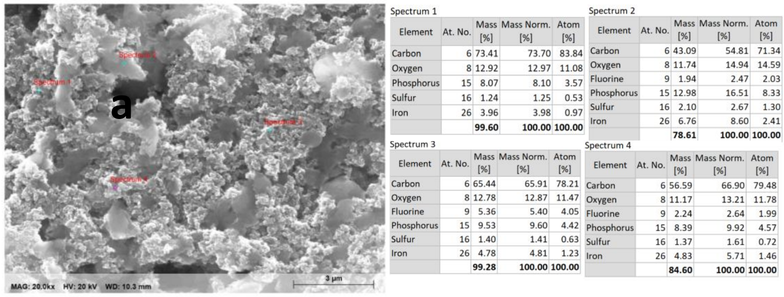

Spectrum 1

Element	At. No.	Mass [%]	Mass Norm. [%]	Atom [%]
Carbon	6	73.41	73.70	83.84
Oxygen	8	12.92	12.97	11.08
Phosphorus	15	8.07	8.10	3.57
Sulfur	16	1.24	1.25	0.53
Iron	26	3.96	3.98	0.97
		**99.60**	**100.00**	**100.00**

Spectrum 2

Element	At. No.	Mass [%]	Mass Norm. [%]	Atom [%]
Carbon	6	43.09	54.81	71.34
Oxygen	8	11.74	14.94	14.59
Fluorine	9	1.94	2.47	2.03
Phosphorus	15	12.98	16.51	8.33
Sulfur	16	2.10	2.67	1.30
Iron	26	6.76	8.60	2.41
		**78.61**	**100.00**	**100.00**

Spectrum 3

Element	At. No.	Mass [%]	Mass Norm. [%]	Atom [%]
Carbon	6	65.44	65.91	78.21
Oxygen	8	12.78	12.87	11.47
Fluorine	9	5.36	5.40	4.05
Phosphorus	15	9.53	9.60	4.42
Sulfur	16	1.40	1.41	0.63
Iron	26	4.78	4.81	1.23
		**99.28**	**100.00**	**100.00**

Spectrum 4

Element	At. No.	Mass [%]	Mass Norm. [%]	Atom [%]
Carbon	6	56.59	66.90	79.48
Oxygen	8	11.17	13.21	11.78
Fluorine	9	2.24	2.64	1.99
Phosphorus	15	8.39	9.92	4.57
Sulfur	16	1.37	1.61	0.72
Iron	26	4.83	5.71	1.46
		**84.60**	**100.00**	**100.00**

Name	Date	Time	HV [kV]	Mag	WD [mm]
Image	7/17/2025	9:16:08 AM	20.0 keV	20000x	10.3 mm

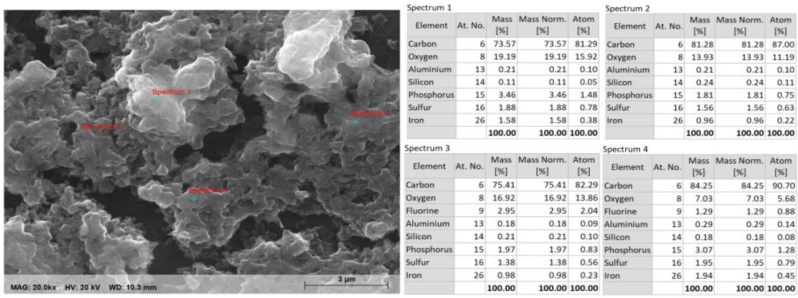

Spectrum 1

Element	At. No.	Mass [%]	Mass Norm. [%]	Atom [%]
Carbon	6	73.57	73.57	81.29
Oxygen	8	19.19	19.19	15.92
Aluminium	13	0.21	0.21	0.10
Silicon	14	0.11	0.11	0.05
Phosphorus	15	3.46	3.46	1.48
Sulfur	16	1.88	1.88	0.78
Iron	26	1.58	1.58	0.38
		**100.00**	**100.00**	**100.00**

Spectrum 2

Element	At. No.	Mass [%]	Mass Norm. [%]	Atom [%]
Carbon	6	81.28	81.28	87.00
Oxygen	8	13.93	13.93	11.19
Aluminium	13	0.21	0.21	0.10
Silicon	14	0.24	0.24	0.11
Phosphorus	15	1.81	1.81	0.75
Sulfur	16	1.56	1.56	0.63
Iron	26	0.96	0.96	0.22
		**100.00**	**100.00**	**100.00**

Spectrum 3

Element	At. No.	Mass [%]	Mass Norm. [%]	Atom [%]
Carbon	6	75.41	75.41	82.29
Oxygen	8	16.92	16.92	13.86
Fluorine	9	2.95	2.95	2.04
Aluminium	13	0.18	0.18	0.09
Silicon	14	0.21	0.21	0.10
Phosphorus	15	1.97	1.97	0.83
Sulfur	16	1.38	1.38	0.56
Iron	26	0.98	0.98	0.23
		**100.00**	**100.00**	**100.00**

Spectrum 4

Element	At. No.	Mass [%]	Mass Norm. [%]	Atom [%]
Carbon	6	84.25	84.25	90.70
Oxygen	8	7.03	7.03	5.68
Fluorine	9	1.29	1.29	0.88
Aluminium	13	0.29	0.29	0.14
Silicon	14	0.18	0.18	0.08
Phosphorus	15	3.07	3.07	1.28
Sulfur	16	1.95	1.95	0.79
Iron	26	1.94	1.94	0.45
		**100.00**	**100.00**	**100.00**

Name	Date	Time	HV [kV]	Mag	WD [mm]
Image	7/17/2025	9:40:53 AM	20.0 keV	20000x	10.3 mm

Figure S9. EDX images of the FeP electrode (a) before and (b) after HER testing.

References

(1) Sharma, S.; Khatri, N.; Puri, S.; Adhikari, M.; Wagle, P.; McIlroy, D. N.; Kalkan, A. K.; Vasquez, Y. Iron Phosphide Nanobundles for Efficient Electrochemical Hydrogen Evolution Reaction in Acidic and Basic Media. *ACS Appl. Mater. Interfaces* **2024**, *16* (45), 61858–61867.

(2) Liu, Q.; Pu, Z.; Asiri, A. M.; Sun, X. Nitrogen-Doped Carbon Nanotube Supported Iron Phosphide Nanocomposites for Highly Active Electrocatalysis of the Hydrogen Evolution Reaction. *Electrochimica Acta* **2014**, *149*, 324–329. https://doi.org/10.1016/j.electacta.2014.10.105.

(3) Jiang, J.; Wang, C.; Zhang, J.; Wang, W.; Zhou, X.; Pan, B.; Tang, K.; Zuo, J.; Yang, Q. Synthesis of FeP2/C Nanohybrids and Their Performance for Hydrogen Evolution Reaction. *J. Mater. Chem. A* **2015**, *3* (2), 499–503.
(4) Guo, X.; Feng, Z.; Lv, Z.; Bu, Y.; Liu, Q.; Zhao, L.; Hao, C.; Li, G.; Lei, Q. Formation of Uniform FeP Hollow Microspheres Assembled by Nanosheets for Efficient Hydrogen Evolution Reaction. *ChemElectroChem* **2017**, *4* (8), 2052–2058. https://doi.org/10.1002/celc.201700366.
(5) Zhu, X.; Liu, M.; Liu, Y.; Chen, R.; Nie, Z.; Li, J.; Yao, S. Carbon-Coated Hollow Mesoporous FeP Microcubes: An Efficient and Stable Electrocatalyst for Hydrogen Evolution. *J. Mater. Chem. A* **2016**, *4* (23), 8974–8977.
(6) Ma, F. X.; Xu, Y. C.; Lyu, F.; Song, B.; Sun, C. S.; Li, Y. Y.; Lu, J.; Zhen, L. Construction of FeP Hollow Nanoparticles Densely Encapsulated in Carbon Nanosheet Frameworks for Efficient and Durable Electrocatalytic Hydrogen Production. *Adv Sci* **2019**, *6*, 1801490.
(7) Xu, Y.; Wu, R.; Zhang, J.; Shi, Y.; Zhang, B. Anion-Exchange Synthesis of Nanoporous FeP Nanosheets as Electrocatalysts for Hydrogen Evolution Reaction. *Chem. Commun.* **2013**, *49* (59), 6656–6658.
(8) Pu, Z.; Amiinu, I. S.; Zhang, C.; Wang, M.; Kou, Z.; Mu, S. Phytic Acid-Derivative Transition Metal Phosphides Encapsulated in N,P-Codoped Carbon: An Efficient and Durable Hydrogen Evolution Electrocatalyst in a Wide pH Range. *Nanoscale* **2017**, *9* (10), 3555–3560.
(9) Lin, C.; Gao, Z.; Yang, J.; Liu, B.; Jin, J. Porous Superstructures Constructed from Ultrafine FeP Nanoparticles for Highly Active and Exceptionally Stable Hydrogen Evolution Reaction. *J. Mater. Chem. A* **2018**, *6* (15), 6387–6392.
(10) Guo, X.; Yu, X.; Feng, Z.; Liang, J.; Li, Q.; Lv, Z.; Liu, B.; Hao, C.; Li, G. Intercalation Synthesis of Prussian Blue Analogue Nanocone and Their Conversion into Fe-Doped CoxP Nanocone for Enhanced Hydrogen Evolution. *ACS Sustain. Chem. Eng.* **2018**, *6* (7), 8150–8158.
(11) Park, Y.; Kang, H.; Hong, Y.; Cho, G.; Choi, M.; Cho, J.; Ha, D.-H. Influence of the Phosphorus Source on Iron Phosphide Nanoparticle Synthesis for Hydrogen Evolution Reaction Catalysis. *ICAE 2019 – Symp. 6 Adv. Mater. Devices Fuel Cell Electrolysis Technol.* **2020**, *45* (57), 32780–32788.
